# Editorial: Finding new hope in old treatments: repurposing immunotherapy in transplantation

**DOI:** 10.3389/fimmu.2025.1738636

**Published:** 2025-11-28

**Authors:** Yeqi Nian, Jasper Iske, Xiaodong Yuan

**Affiliations:** 1 Department of Kidney Transplantation, Tianjin First Central Hospital, School of Basic Medical Sciences, Tianjin Medical University, Tianjin, China; 2 Department of Cardiothoracic and Vascular Surgery, Deutsches Herzzentrum der Charité (DHZC), Berlin, Germany; 3 Department of Urology, Renji Hospital, School of Medicine, Shanghai Jiaotong University, Shanghai, China

**Keywords:** transplantation, immunity, drug repurposing, immunotherapy, transplant rejection

Organ transplantation has transformed the management of end-stage organ failure; however, long-term graft survival continues to be limited by persistent obstacles such as ischemia-reperfusion injury, refractory rejection, chronic allograft dysfunction, and *de novo* malignancies. Although the introduction of immunosuppressive agents like tacrolimus and cyclosporine in the 1970s represented a major advance, current therapeutic regimens remain insufficient to fully resolve these complex issues, highlighting the ongoing need for innovative approaches (1). Drug repurposing—the application of existing drugs to new clinical indications—has emerged as a promising strategy to accelerate therapeutic discovery in transplantation. By harnessing immunomodulatory agents already approved for oncology and autoimmune conditions, this approach bypasses lengthy drug development pathways and offers novel solutions for immune dysregulation following transplantation (2). This Research Topic, “Finding New Hope in Old Treatments: Repurposing Immunotherapy in Transplantation,” explores the immunological intersections between transplantation, cancer, and autoimmunity, and illustrates how repurposed drugs may help overcome critical barriers in clinical practice.

A fundamental requirement for successful drug repurposing is a deep understanding of shared immune mechanisms. Several articles in this Topic delve into such pathways. The review by Xu et al. elucidates how chemokine networks—central to both autoimmunity and cancer—drive alloreactive T-cell trafficking in graft-versus-host disease, highlighting potential targets for therapeutic intervention. Similarly, Wang et al. examine the role of innate immune signaling, a key contributor to sterile inflammation, in orchestrating oxidative stress and cell death following transplantation, positioning TLR4 as a viable target for mitigating early graft injury. Complementing these insights, Lu et al. employ bioinformatics approaches to bridge concepts from onco-immunology, identifying immunogenic cell death-related genes as diagnostic biomarkers and reinforcing the molecular overlap between tissue damage and immune activation.

Translating these mechanistic insights into therapeutic strategies represents a crucial next step. Hu et al. exemplify the repurposing paradigm by defining a T-cell-mediated rejection (TCMR)-associated cytokine gene signature and screening drug databases to identify the PPARγ agonist rosiglitazone—an antidiabetic agent—as a potent suppressor of T-cell activation, demonstrating its synergistic efficacy with rapamycin in prolonging graft survival. This integrated computational and experimental pipeline underscores the power of systematic repurposing. In parallel, Zhang et al. investigate glycine transporter 1 (GlyT1) as a metabolic checkpoint, revealing its role in Th1 differentiation and proposing ALX-5407 as a novel agent that attenuates alloimmune responses when combined with standard therapy.

Clinical applications and patient-specific scenarios further bring the promise of repurposing to life. A recent clinical trial provides compelling evidence supporting the repurposing of the anti-CD38 antibody felzartamab—originally developed for multiple myeloma—for the depletion of plasma cells and mitigation of antibody-mediated rejection, with a favorable safety profile (3). In the setting of transplant complications, Xiong et al. report the successful off-label use of baricitinib, a JAK1/2 inhibitor approved for rheumatoid arthritis, to manage life-threatening steroid-refractory chronic graft-versus-host disease, offering a new therapeutic avenue for this challenging condition. Furthermore, Kong et al. review the pressing need for agents that provide both immunosuppressive and anti-tumor effects, discussing the dual potential of mTOR inhibitors and chemotherapeutic drugs such as capecitabine in transplant recipients with oncological risk factors.

The development of non-genotoxic conditioning regimens is also critical, particularly in hematopoietic stem cell transplantation. Okalova et al. discuss how monoclonal antibodies and antibody–drug conjugates may replace traditional alkylating agents or irradiation, potentially reducing the risks of secondary malignancies and organ toxicity.

The collective work in this Topic underscores that drug repurposing is not merely about finding new uses for old drugs; it is a strategic inquiry into the universality of immunological principles. By mining the rich landscape of approved immunotherapies, the transplant community can accelerate the development of precise, effective, and safer regimens. The studies presented here—spanning bioinformatics discovery, mechanistic validation in models, and proof-of-concept clinical trials—chart a course for this integrated future. We hope this Research Topic inspires continued collaboration across immunology, oncology, and computational biology to overcome the enduring barriers in transplantation and improve long-term outcomes for recipients worldwide.
